# Three-Dimensional Aggregated Spheroid Model of Hepatocellular Carcinoma Using a 96-Pillar/Well Plate

**DOI:** 10.3390/molecules26164949

**Published:** 2021-08-16

**Authors:** Sang-Yun Lee, Yvonne Teng, Miseol Son, Bosung Ku, Hyun Ju Hwang, Vinay Tergaonkar, Pierce Kah-Hoe Chow, Dong Woo Lee, Do-Hyun Nam

**Affiliations:** 1 Department of Health Sciences and Technology, Samsung Advanced Institute for Health Sciences and Technology (SAIHST), Sungkyunkwan University, Seoul 06351, Korea; leesangyun316@gmail.com; 2 Central R & D Center, Medical & Bio Decision (MBD) Co., Ltd., Suwon 16229, Korea; goos4684@gmail.com (B.K.); 11pluto15@naver.com (H.J.H.); 3 Research & Development Department, AVATAMED Pte. Ltd., Singapore 117408, Singapore; yvonne.teng@avatamed.com (Y.T.); miseol.son@avatamed.com (M.S.); 4 Laboratory of NFκB Signaling, Institute of Molecular and Cell Biology (IMCB), A*STAR (Agency for Science, Technology and Research), Singapore 138673, Singapore; vinayt@imcb.a-star.edu.sg; 5 Department of Pathology, Yong Loo Lin School of Medicine, National University of Singapore (NUS), Singapore 117597, Singapore; 6 Division of Surgery and Surgical Oncology, Singapore General Hospital (SGH) and National Cancer Centre Singapore (NCCS), Singapore 169610, Singapore; pierce.chow.k.h@singhealth.com.sg; 7 Department of Hepatopancreatobiliary and Transplant Surgery, Singapore General Hospital (SGH), Singapore 169608, Singapore; 8 Surgery Academic Clinical Programme, Duke-NUS Medical School, Singapore 169857, Singapore; 9 Genome Institute of Singapore (GIS), Singapore 138672, Singapore; 10 Department of Biomedical Engineering, Konyang University, Daejeon 35365, Korea; 11 Department of Neurosurgery, Samsung Medical Center, Sungkyunkwan University School of Medicine, Seoul 06351, Korea

**Keywords:** 3D cell culture, hepatocellular carcinoma cell line, in vitro extracellular matrix remodeling, cancer spheroids in Matrigel, high-throughput screening, 96-pillar/well plate

## Abstract

A common method of three-dimensional (3D) cell cultures is embedding single cells in Matrigel. Separated cells in Matrigel migrate or grow to form spheroids but lack cell-to-cell interaction, which causes difficulty or delay in forming mature spheroids. To address this issue, we proposed a 3D aggregated spheroid model (ASM) to create large single spheroids by aggregating cells in Matrigel attached to the surface of 96-pillar plates. Before gelling the Matrigel, we placed the pillar inserts into blank wells where gravity allowed the cells to gather at the curved end. In a drug screening assay, the ASM with Hepatocellular carcinoma (HCC) cell lines showed higher drug resistance compared to both a conventional spheroid model (CSM) and a two-dimensional (2D) cell culture model. With protein expression, cytokine activation, and penetration analysis, the ASM showed higher expression of cancer markers associated with proliferation (p-AKT, p-Erk), tight junction formation (Fibronectin, ZO-1, Occludin), and epithelial cell identity (E-cadherin) in HCC cells. Furthermore, cytokine factors were increased, which were associated with immune cell recruitment/activation (MIF-3α), extracellular matrix regulation (TIMP-2), cancer interaction (IL-8, TGF-β2), and angiogenesis regulation (VEGF-A). Compared to CSM, the ASM also showed limited drug penetration in doxorubicin, which appears in tissues in vivo. Thus, the proposed ASM better recapitulated the tumor microenvironment and can provide for more instructive data during in vitro drug screening assays of tumor cells and improved prediction of efficacious drugs in HCC patients.

## 1. Introduction

Hepatocellular carcinoma (HCC) is the sixth most common cancer and the third most frequent cause of cancer-related mortality worldwide [1,2,3]. The heterogeneous malignancy with complex carcinogenesis of HCC is a major barrier to the study of tumorigenesis and investigating molecular therapeutic targets and diagnostic biomarkers for HCC prevention. HCC patients typically have poor tolerance of systemic chemotherapy due to underlying chronically damaged tissue and liver dysfunction that contain considerable inflammation, fibrosis, and cirrhosis. Therefore, to find the most responsive and effective drug for HCC patients, many preclinical studies and in vitro drug assays are conducted under two-dimensional (2D) monolayer cell culture conditions. The conventional 2D cell culture system is well established and generates highly reproducible data. As a result, high-throughput screening (HTS) methods have been widely used for scientific experimentation. However, in the field of drug discovery, the limitations of HTS have been increasingly recognized. The conventional 2D cell culture-based HTS platform does not reflect the actual patient’s tumor microenvironment (TME), such as physiological cell-to-cell and cell-to-extracellular matrix (ECM) interactions [4]. Additionally, many cells lose some of their phenotypic and genomic properties when grown using in vitro methods with 2D cell culture. As a result of these limitations, experimental results using 2D cell culture may provide ambiguous and nonpredictive data for in vivo and clinical responses [5,6,7].

One model that may overcome issues with 2D cell culture is the three-dimensional (3D) culture model, which was devised to better mimic the in vivo behavior of tumor cells under more adaptable conditions. A recent study reported that 3D cultured spheroids composed of patient-derived tumor cells could maintain tumor characteristics in vitro for an extended period [8]. These biological and physical aspects of 3D cell cultures affect not only the expression of the cell surface receptors but also induce the activation of signal transduction from the outside to the inside of cells. It has been reported that 3D cultured cell responses are more similar to in vivo testing results compared to those from 2D cell culture [9,10]. Our group also developed conventional spheroid model (CSM) as previously reported [11,12,13,14,15,16].

**Figure 1:**
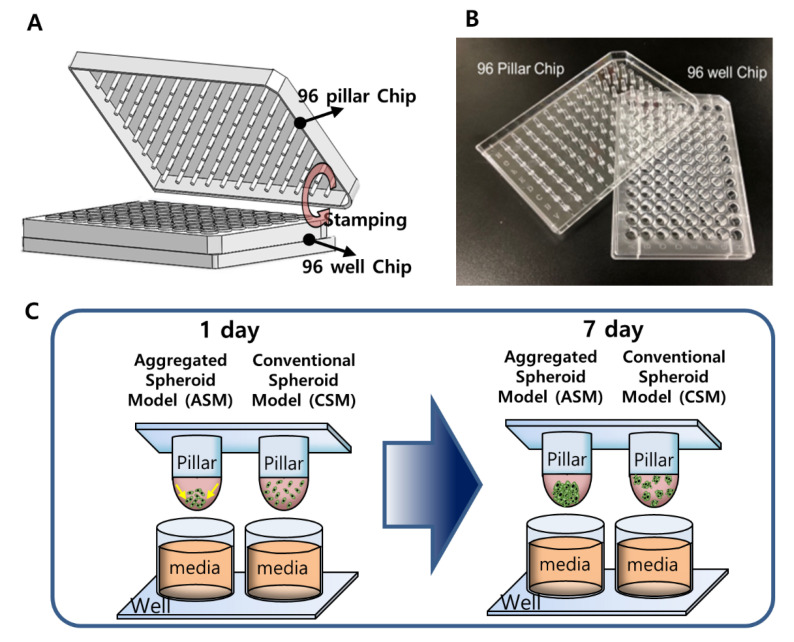
The 3D-HCC cells were cultured three-dimensionally with six different drugs in a 96-pillar/well plate (seven doses and three replicates). (**A**) Schematic view of the 96-pillar/well plate for the 3D-HCC cell-based HTS. (**B**) Image of fabricated 96-pillar/well plate. (**C**) Concept illustration of ASM compared to CSM.

Among ECM-embedded 3D cell cultures, Matrigel is the most used. Matrigel is a soluble extract of basement membrane proteins derived from the Engelbreth–Holm–Swarm tumor that forms a 3D gel at 37 °C. It is known to enhance biological features, reflecting actual cancer cell malignancy, and is related to cell proliferation, differentiation, morphology, gene and protein expression, and cellular responses to external stimuli. Thus, the 3D cell culture of HCC typically uses the Matrigel for 3D cell culture. In conventional Matrigel-embedded 3D cell culture, HCC cells were evenly located in Matrigel and form multi-spheroids. However, our group found that the morphology and number of spheroids were dramatically affected by how the HCC cells were initially located when the Matrigel was gelled. If the initial cells in Matrigel were close to each other, those conditions can produce large, single spheroids, as shown in Figure 1. The aggregated spheroid model (ASM) occurs when two HCC cells (Hep3B and HepG2) are gathered at the curved end of the Matrigel because of gravity before gelling on the pillar chip, and HCC cells form large single spheroids. In the current paper, the ASM was analyzed for drug sensitivity, protein expression, cytokine activation, and drug penetration compared to the conventional spheroid model (CSM) and 2D cell culture.

## 2. Results and Discussion

### 2.1. HTS of Six Anti-Cancer Drug Responses in Each 3D-Cell Model

We selected drugs that are commonly used for HCC patients in the clinics, particularly drugs targeting vascular endothelial growth factor receptor (VEGFR), tyrosine-protein kinase Met (c-Met), mitogen-activated protein kinases/extracellular signal-regulated kinases (Ras/Raf/MEK/ERK), platelet-derived growth factor receptor-β (PDGFR-β), and those that cause DNA/RNA synthesis damage, etc. All drugs were in phase III or IV trials or were approved oncology drugs from public data provided by the US Food and Drug Administration (FDA). The six drugs selected (including three controls) were dispensed into 96-well plates in a dose-dependent manner.

**Figure 2:**
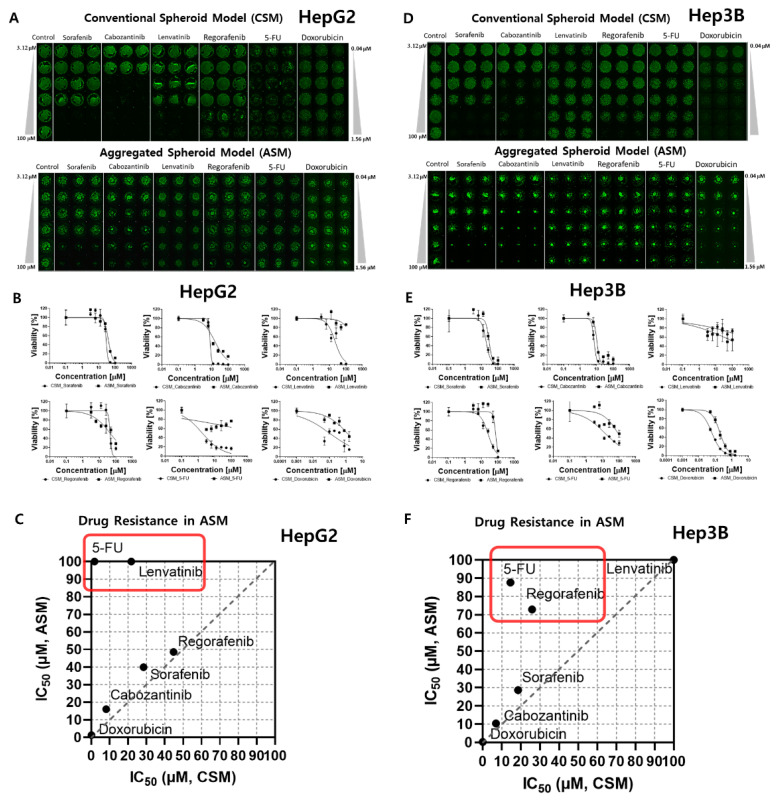
Comparative dose–response curve analysis of six different drugs in 3D cell models. The IC_50_ was calculated to compare the 3D live-cell staining dye expression level according to the dose of each of the six drugs. (**A**) Combined image of 3D live HepG2 cells in the CSM and ASM. (**B**) Dose–response curves and a summary table of quantitatively analyzed drug efficacy according to the doses of the six drugs tested in the HepG2 CSM and ASM. (**C**) IC_50_ comparison of ASM and CSM in HepG2. 5-FU and Lenvatinib showed higher drug resistance in the ASM than CSM. (**D**) Combined image of 3D live Hep3B cells in the CSM and ASM. (**E**) Dose–response curves and a summary table of quantitatively analyzed drug efficacy according to the doses of the six drugs tested in the Hep3B CSM and ASM. (**F**) IC_50_ comparison of ASM and CSM in Hep3B. 5-FU and Regorafenib showed higher drug resistance in the ASM than CSM.

3D models of CSM and ASM show higher drug resistance than 2D models, as shown in Table 1. Especially, six drugs indicated higher drug resistance in the ASM than in the CSM. 5-FU (5-Fluorouracil) and Lenvatinib show higher resistance in ASM, as shown in Figure 2C,F. When HepG2 cells were treated with 5-FU, the IC_50_ values of the CSM and ASM were confirmed to be 1.78 µM and more than 100 µM, respectively, as shown in Figure 2A–C. We found that 5-FU targeting DNA/RNA synthesis inhibition drugs severely inhibited the HepG2 cell viability in 2D HTS and CSM conditions. Conversely, the ASM showed no response based on IC_50_ values at 100 µM for the 5-FU drug treatment (Table 1 and Figure 2C). In addition, when Hep3B cells were treated with 5-FU, the ASM showed approximately six times higher drug resistance compared to the CSM. The IC_50_ values of the CSM and ASM were confirmed to be 14.5 µM and 87.6 µM, respectively, as shown in Figure 2D–F. The current study demonstrated that HepG2 and Hep3B cells acquire substantial resistance to 5-FU when the cell-to-cell and cell-to-ECM interactions are reestablished in ASM.

Lenvatinib, an inhibitor of VEGFR 1–3, fibroblast growth factor receptor (FGFR) 1–4, PDGFR α, RET, and KIT, showed differences in drug responses when comparing the 2D HTS and 3D cell model results (Table 1). When HepG2 cells were treated with Lenvatinib, the IC_50_ values of the CSM and ASM were confirmed to be 21.77 µM and over 100 µM, respectively, as shown in Figure 2A–C. Lenvatinib dramatically inhibited the Hep3B cell viabilities in 2D HTS conditions under the 1.4 µM IC_50_ value as shown in Table 1. However, in the 3D cell models, CSM and ASM, Lenvatinib showed no response, and IC_50_ values measured over 100 µM (Figure 2D,E). Through these experiments, we found that the ASM showed a drug-resistant response, and these additional ECM compositions may influence drug response through altered local drug availability, by affecting the expression of drug targets, or by changing intrinsic cellular defense mechanisms such as increased DNA damage repair or evasion of apoptosis [17].

### 2.2. Western Blot Analysis of Epithelial Cell Markers, Cell Proliferation Receptor, and ECM/Cell Tight Junction Protein Expression

We conducted a molecular analysis related to original epithelial characteristics and the different drug responses of the two 3D HCC models (ASM, CSM) and 2D model.

**Figure 3:**
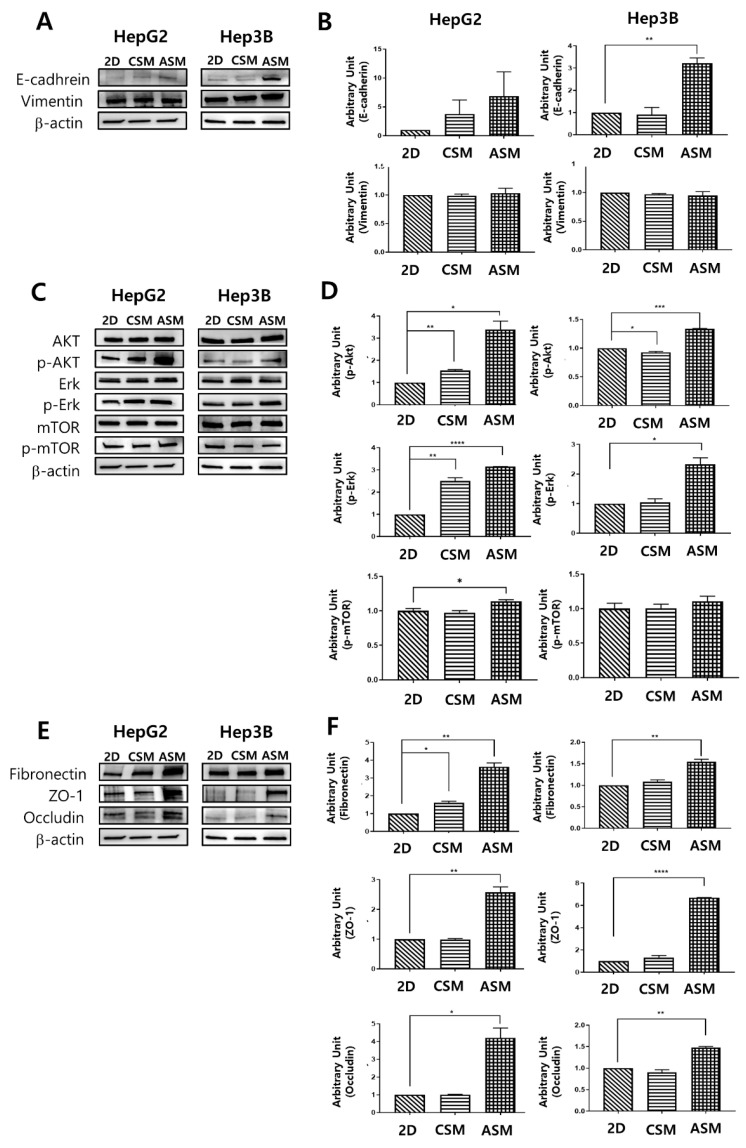
Western blot analysis of epithelial cell protein and cell proliferation receptor protein expression. Changes in the expression level of the epithelial cell protein (**A**,**B**), proliferation receptor protein (**C**,**D**), ECM/ Cell tight junction protein (**E**,**F**) as determined by western blot analysis. Increased expression of E-cadherin, p-AKT, p-Erk, Fibronectin, ZO-1, and Occludin under ASM culture conditions. All factors were normalized to β-actin. * *p* < 0.05, ** *p* < 0.01, *** *p* < 0.001, **** *p* < 0.0001.

As shown in Figure 3, we performed western blot analysis by recovering cells cultured in 2D and 3D cell models (CSM and ASM). Since HCC cells have epithelial properties, it can be assumed that culture conditions in which epithelial markers are well expressed can maintain the actual tumor characteristics. HepG2 and Hep3B were both classified as epithelial based on their expression of E-cadherin protein and mRNA [18]. The epithelial characteristics of HCC cells were not well maintained in the 2D cell culture and the CSM. Conversely, in the ASM, the expression of E-cadherin, an epithelial marker, was relatively high. E-cadherin was overexpressed in the ASM. HepG2 and Hep3B had E-cadherin values of 6.88 and 3.21, respectively (Figure 3A,B). These amounts were greater than those found in the 2D and CSM cell culture models.

Phosphorylated-AKT (p-AKT) and phosphorylated-Erk (p-Erk) activities, which are known to be related to cell proliferation and important components of cell survival pathways [19], had the greatest increase in the ASM model compared to the 2D and CSM cell culture models (Figure 3C,D). p-AKT was overexpressed in the ASM in HepG2 and Hep3B. Similarly, The ASM of HepG2 and Hep3B cells had higher levels of p-Erk expression. However, p-mTOR expression in the ASM of HepG2 and Hep3B cells was similar or slightly increased. Therefore, when culturing HCC cells in the ASM, HCC cells can maintain and display the proliferative characteristics of more malignant tumors, which could affect the high drug resistance of the ASM.

The expression levels of the ECM component (Fibronectin) and structure of tight junctions (ZO-1, Occludin) were measured by western blot analysis to understand the differences of the drug response in each cell culture model. Fibronectin, a high-molecular-weight glycoprotein of ECM, was overexpressed in the ASM. It has been reported that Fibronectin was observed in drug-resistant tumor tissues [20]. Fibronectin production occurred in the actual TME and appeared to promote drug resistance [21,22,23]. Additionally, Fibronectin plays a crucial role in growth, differentiation, adhesion, and migration [24]. In case of tight junctions, ASM has high expression levels of ZO-1 and Occludin, the structures of tight junctions, making drug penetration difficult. Previously ZO-1 alternation made the different drug resistance [25]. Therefore, the overexpression of tight junction markers, ZO-1 and Occludin, allows for increased tight junction function and decreased drug delivery into cancer cells. The ASM of HepG2 and Hep3B cells had higher levels of Fibronectin, ZO-1 and Occludin expression when normalized to β-actin in the ASM where HepG2 and Hep3B had values of 3.63 and 1.55, 2.58 and 6.69, 4.21 and 1.48, respectively. These values were greater than those in the 2D and CSM (Figure 3E,F).

Given these findings, we confirmed that the ASM has the advantage of maintaining the actual HCC cells epithelial characteristics more efficiently than the conventional 2D cell culture, the CSM. The ASM showed the increased expression of protein markers and secretion of ECM associated with cancer cell proliferation and drug resistance.

### 2.3. High Content Image Analysis of Epithelial Cell Markers, and ECM/Cell Tight Junction Protein Expression

**Figure 4:**
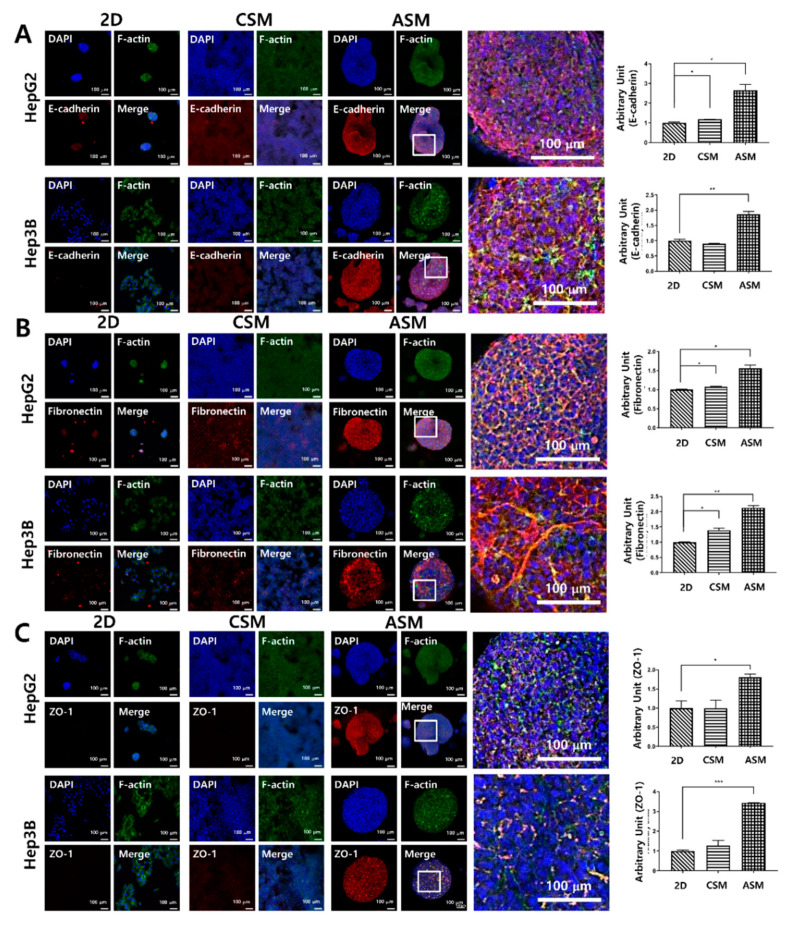
High-content image analysis of epithelial cancer cell protein and drug penetration-related protein expression. The expression of E-cadherin (**A**), Fibronectin (**B**), and ZO-1 (**C**) increased under ASM culture conditions. Optical sections were acquired at 10 µm intervals and stacked into a Z-projection. Scale bars: 100 µm * *p* < 0.05, ** *p* < 0.01, *** *p* < 0.001.

We conducted immunofluorescence analysis for E-cadherin, an epithelial cancer cell marker, Fibronectin, an ECM marker, and ZO-1, a tight junction marker, to cross-validate the western blot analysis results. The epithelial characteristics of HCC cells were not well maintained in the 2D cell culture and the CSM. However, in the ASM, E-cadherin was overexpressed when normalized to DAPI, where HepG2 and Hep3B had values of 2.66 and 1.86, respectively. These levels were greater than those in the corresponding 2D and CSM cell culture model counterparts, as shown in Figure 4A.

Fibronectin and ZO-1 were not well expressed in the 2D cell culture and CSM. However, in the ASM, these components related to drug resistance were well maintained. Fibronectin was overexpressed when normalized to DAPI, where HepG2 and Hep3B had values of 1.56 and 2.13, respectively. Additionally, the expression level of ZO-1 was measured as 1.81 and 3.44 in HepG2 and Hep3B, respectively. These levels were greater than those in the corresponding 2D and CSM cell culture model counterparts, as shown in Figure 4B,C. Thus, we confirmed that the epithelial characteristics of HCC cells were better maintained, and the expression of Fibronectin and tight junction proteins related to drug resistance was higher in the ASM. This is related to high drug resistance to six anticancer drugs when HCC cells are cultured in the ASM, suggesting the ASM is superior to the conventional 2D cell culture-based drug testing assay.

### 2.4. Cytokine Secretion Analysis Related to Cell Proliferation and Drug Resistance

**Figure 5:**
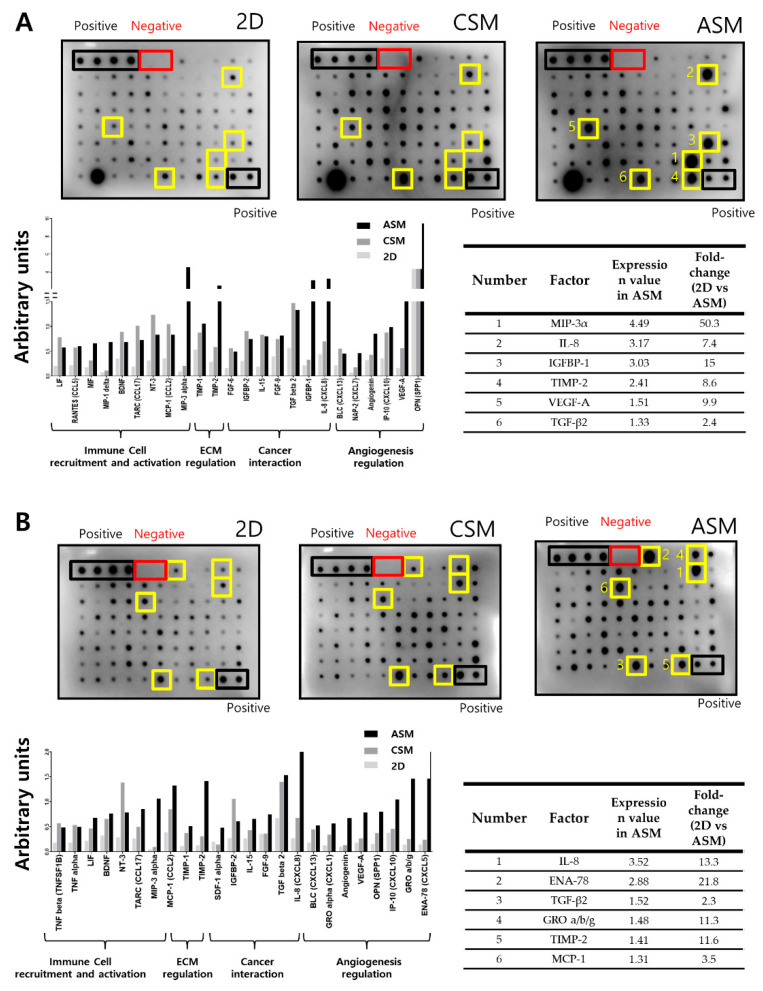
Cell proliferation and drug resistance-related cytokine secretion analysis. Increased secretion of chemokines and cytokines in conditioned media (CM) of ASM culture condition. Representative images of human cytokine array analysis in CM of HepG2 (**A**) and Hep3B (**B**). The graph indicates factors showing increases to the top 30% in the ASM. Six factors were detected to be the highest under ASM culture conditions (yellow boxes).

Secreted cytokines were analyzed to understand differences in drug resistance and phenotypic characteristics of the two HCC cancer cells according to the 2D and 3D cell models (Figure 5).

We recovered the cell culture medium in 2D cell culture and 3D cell models (CSM and ASM) and quantitatively analyzed the secreted cytokines. Among the secreted cytokines, those corresponding to the top 30% in the ASM were selected. These cytokines had more than a twofold increase when compared to the 2D cell culture condition, as shown in Figure 5. Tissue inhibitors of metalloproteinases-2 (TIMP-2) among ECM regulation marker, Interleukin (IL)-8/ Transforming growth factor-β (TGF-β) among cancer interaction marker were overexpress in ASM compared to CSM. HepG2 cell-to-cell interactions in the ASM stimulated cancer cell interaction, angiogenesis, ECM regulation, and immune cell recruitment/activation. Paracrine signaling of IL-8, IL-15, TGF-β, VEGF-A, Chemokine ligand 2 (CCL2), CCL5, CCL17, Chemokine (C-X-C motif) ligand 7 (CXCL7), CXCL8, CXCL10, CXCL13, Insulin-like growth factor-binding protein-1 (IGFBP-1), IGFBP-2, osteopontin (OPN), angiogenin, TIMP-1, and TIMP-2 was increased in the ASM when compared to 2D and CSM conditions (Figure 5A). Hep3B cells cultured in ASM stimulate cancer cell interaction, angiogenesis, ECM regulation, immune cell recruitment, and activation. Paracrine signaling of IL-8, IL-15, TGF-β, VEGF-A, CCL2, CCL17, CXCL1, CXCL5, CXCL13, IGFBP-2, OPN, angiogenin, TIMP-1, TIMP-2, stromal cell-derived factor 1 (SDF-1), tumor necrosis factor-a (TNF-α), and TNF-β was increased in the ASM when compared to the 2D and CSM conditions (Figure 5B). When HCC cells were cultured in the ASM, cytokine secretion similar to the TME of actual cancer patients was occurred, which means that the ASM can be a suitable method to describe the in vivo-like TME.

### 2.5. Drug penetration Assay for Validation of Drug Resistance in the ASM Using an Auto-Fluorescent Drug

**Figure 6:**
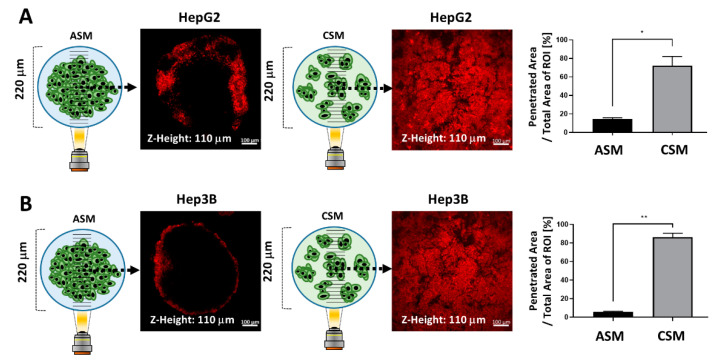
Drug penetration assay for validation of drug resistance in the ASM using the auto-fluorescent drug (DOX). (**A**) Images of DOX penetration in HepG2 ASM and CSM and quantitatively analyzed drug penetration ratios in each 3D model. The penetrated DOX fluorescence was calculated at 14.69% and 72.2%, respectively. (**B**) Images of DOX penetration in HepG2 ASM and CSM and quantitatively analyzed drug penetration ratios in each 3D model. The penetrated DOX fluorescence was calculated at 5.75% and 86.37%, respectively. * *p* < 0.05, ** *p* < 0.01.

We investigated the penetration behavior of Doxorubicin (DOX) in CSM and ASM, as shown in Figure 6. DOX, which is widely used in clinical practice and has its own orange-red fluorescence, was chosen as a low-molecular-weight antitumor agent. We treated the cells with DOX for 12 h at a dose of 300 µM. Representative spheroid cross-sections observed by confocal microscopy are shown in Figure 6. The average penetrated areas where the drug fluorescence signals are detectable and at the middle Z-position of 3D cancer spheroids were normalized by the total area of region of interest (ROI).

The penetrated DOX fluorescence was detected at 14.69% and 72.2% in the HepG2 ASM and CSM respectively (Figure 6A). In the Hep3B ASM and CSM, the penetrated DOX fluorescence was detected at 5.75% and 86.37%, respectively, as shown in Figure 6B. As shown in the previous results, in the ASM, it is difficult for the drug to penetrate into the cancer spheroid. Since this is a 3D structural feature similar to that of actual cancer tissue, the ASM can be a better drug response analysis model than the conventional 2D cell culture-based in vitro assay.

## 3. Materials and Methods

### 3.1. Preparation of the 96 Pillar/Well Plates

The 96-pillar/well plate was manufactured by plastic injection molding and is a robust and flexible platform for mammalian cell cultures, enzymatic reactions, viral infections, and compound screenings (Figure 1). The 96-pillar plate is made of polystyrene and contains 96-pillars (with a 2-mm pillar diameter and 9-mm pillar-to-pillar distance). The 96-pillar plate was coated with poly-L-lysine solution to support Matrigel attachment on the hydrophilic pillar surface. The plate has 96 complementary wells with a 7-mm well diameter and 9-mm well-to-well distance, and it can be combined or “stamped” with a 96-pillar plate to conduct the 3D cell culture and the drug efficacy test. Plastic molding was performed with an injection molder (Sodic Plustech Inc., Schaumburg, IL, USA).

### 3.2. Experimental Procedure of High-Throughput Drug Screening

For HTS and implementation of 3D cell culture models, 1.5 µL volume of mixtures containing approximately 4000 cells and 50% Matrigel were automatically dispensed onto the 96-pillar plate surface using an ASFA™ Spotter ST (Medical & Bio Decision, Suwon, South Korea). The ASFA™ Spotter ST uses a solenoid valve (The Lee Company, Westbrook, CT, USA) to dispense 1.5 µL droplets of the cell–Matrigel mixture on the 96-pillar plate surface.

**Figure 7:**
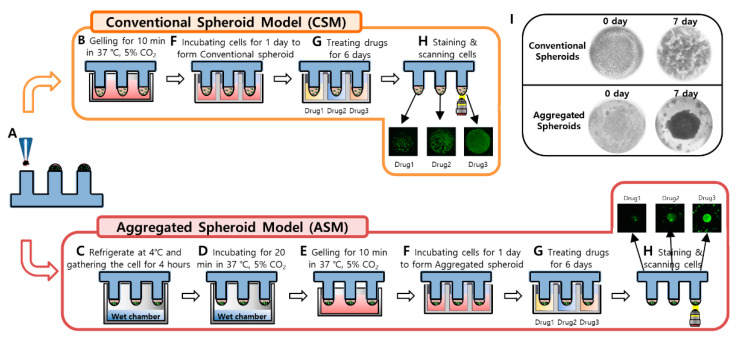
Experimental procedures of 3D Cell culture model implementation (CSM and ASM) using a 96-pillar/well plate. (**A**) Cell and Matrigel mixture dispensed on the surface of the 96-pillar plate. (**B**) 3D cell and Matrigel mixture directly gelling after dispensing step. (**C**) Cell down and aggregate in the core of Matrigel at 4 °C before gelling the Matrigel (**D**) 3D cell and Matrigel gelation step with the pillar surface facing downward. (**E**) Additional gelling step for 10 min. (**F**) One-day incubation for stabilizing the cells by stamping the 96-pillar plate into the 96-well plate filled with cell culture medium. (**G**) Drug exposure by replacing the 96-well plate with another filled with six different drugs. (**H**) Green fluorescence image scanning after 3D live-cell staining. (**I**) Representative images for spread 3D cells in the CSM and aggregated 3D cells in the ASM.

After dispensing the cells, two types of 3D cell culture models were implemented (CSM and ASM), as shown in Figure 7A. To implement the CSM, directly after gelling the cell and Matrigel combination were dispensed (Figure 7B). To implement the ASM, the gathering cells and Matrigel gelation steps were carried out with the pillar surface facing down (Figure 7C,D). The 96-pillar plate was combined with the pre-warmed culture medium and subjected to additional gelling for 10 min (Figure 7E). The 96-pillar plate containing HCC cells in Matrigel was sandwiched or “stamped” with the 96-well plate for 3D cell culture and incubated for 1 day to form spheroids (Figure 7F). Each plate was comprised of 96 wells, which contained 200 µL of cell culture medium. For the CSM condition, after the cell-Matrigel mixture was stabilized using each gelation method, 3D cells were observed with an optical microscope to confirm they were evenly spread in Matrigel. In the ASM condition, it was confirmed that cells were aggregated in the center of the pillar surface, as shown in Figure 7I.

Cells were stabilized in the Matrigel spots before being treated with drugs. After stabilizing cells, viability was checked by 3D live-cell staining using Calcein AM dye (Invitrogen, Carlsbad, CA, USA). The majority of cells were alive based on the level of green fluorescence as shown in Figure 7H. To analyze the efficacy of all six drugs, we designed a dose–response curve. The 96-well plate was divided into seven regions. Each region was composed of a 3 × 7 well array corresponding to six different drug doses (including one control) and their triplicates. After treatment with the six different drugs for six days, drug response was analyzed through viability quantification of 3D live-cell staining images. As shown in Figure 7H, the area value of 3D live-cells differed depending on the drug response.

To determine a robust and reproducible assay, a means coefficient of variation (CV) value of less than 10% is used for a good cell-based assay. [26] When analyzing the CV value of the CSM model using the 96-pillar plate for 3D cell culture of this study, the CV value was 2.92% after six days of culture, showing high dispensing uniformity and reproducibility, as shown in Figure S1A. Similarly, the ASM model had a CV value of 7.89%, which was confirmed to be of suitable quality for high-throughput screening analysis for drug discovery, as shown in Figure S1B.

### 3.3. Experimental Procedure for Image Scanning and Data Analysis

An automatic optical fluorescence scanner (ASFA™ Scanner HE, Medical & Bio Decision, Suwon, South Korea) was used to measure green fluorescence intensities (excitation/emission, 494/517 nm to lasers) on the 96-pillar surface using an 8-bit code among the RGB codes (0–255), as shown in Figure 7H. The ASFA Ez SW (Medical & Bio Device) was used to calculate the total and average green areas of the scanned images of cultured cells from each 3D cell culture model. To determine drug efficacy, dose–response curves were plotted using the normalized 3D cell viability values according to the dose of the drugs (GraphPad Prism 8, GraphPad Software, San Diego, CA, USA). The half-maximal inhibitory concentration (IC_50_) values were calculated automatically in the XY analysis completed with the GraphPad Prism 8 software.

### 3.4. Cell Culture

Human HCC cell lines Hep3B and HepG2 were purchased from the Korean Cell Line Bank (Seoul, South Korea). Both Hep3B and HepG2 were cultured in DMEM medium (Gibco, Grand Island, NY, USA) with 100 µg/mL of streptomycin, 100 units/mL of penicillin, 250 ng/mL of amphotericin B, and 10% fetal bovine serum. Cell lines were maintained at 37 °C in a 5% CO_2_-humidified atmosphere and passaged every four days. Typically, we use the Hep3B and HepG2 cell lines under 20 passages after thawing the frozen cell stock. Under 20 passages, we observed that the Hep3B and HepG2 cell lines easily formed 3D cells in 50% Matrigel on the chip platform.

### 3.5. Drugs Preparation

Two 3D-HCC cancer in vitro models (CSM and ASM) were used in drug sensitivity assays to identify changes in drug sensitivity for each model. The drugs most commonly used for HCC were selected. Sorafenib (S7397), Cabozantinib (S1119), Lenvatinib (S1164), Regorafenib (S1178), 5-FU (S1209), and DOX (S1208) were purchased from Selleckchem (Houston, TX, USA). Drugs were dissolved in a stock solution of dimethyl sulfoxide (DMSO, 100 mM).

### 3.6. 3D-Cell Viability Assay

Hep3B and HepG2 cells were seeded in a 96-pillar tray at a density of 4000 cells per pillar in triplicate for each treatment. One day after seeding, cells were treated with drugs in a two-fold and seven dose point serial dilution from 100 µM to 3.12 µM. After six days of incubation at 37 °C in a 5%-CO_2_ humidified incubator, cell viability was determined using an adenosine triphosphate (ATP) monitoring system based on firefly luciferase (CellTiter-Glo^®^ 3D Cell Viability Assay, Promega, Madison, WI, USA) and 3D live-cell staining (Calcein AM, Invitrogen, Carlsbad, CA, USA), according to manufacturer’s protocol. Briefly, the assay mixture was prepared in an ATP monitoring system by adding 20 µL of CellTiter-Glo^®^ 3D reagent into 60 µL of medium per well. Cells were lysed with an assay mixture by shaking for 5 min, followed by incubation for 25 min at room temperature. Viable cells were estimated using the SpectraMax iD3 Reader (Molecular Devices LLC, San Jose, CA, USA). For 3D live-cell staining, the staining solution was prepared by adding 1 µL of Calcein AM into 7 mL of DMEM medium. Cells were incubated with staining solution for 1 h at 37 °C in a 5% CO_2_-humidified atmosphere. Live-cell images were acquired using an automatic fluorescence microscope scanner (ASTA Scanner™, Medical & Bio Decision, Suwon, South Korea)

### 3.7. Western Blot Analysis

Total cell lysates were prepared using a cOmplete™ Lysis-M buffer solution (Roche Life Science, Penzberg, Germany) from the Hep3B and HepG2 hepatocellular carcinoma cell lines. Total protein in lysates was quantified using the Bio-Rad Protein Assay Dye Reagent Concentrate (Bio-Rad, Hercules, CA, USA), 10 μg of total protein was loaded onto 4–20% Mini-PROTEAN TGX™ Precast Protein Gels (Bio-Rad, Hercules, CA, USA) and transferred onto iBlot^®^ PVDF gel Transfer Stack membranes (Thermofisher Scientific, Waltham, MA, USA). The membranes were blocked with 5% bovine serum albumin (BSA) in Tris-buffered saline containing 0.05% Tween-20 at room temperature, and incubated with antibodies against AKT (1:1000, Cell Signaling Technology (CST), #4970), p-AKT (Ser473) (1:1000, CST, #4060), Erk 1/2 (1:1000, CST, #4695), p-Erk (Thr202/Tyr204) (1:1000, CST, #4370), mTOR (1:1000, CST, #2983), phosphorylated-mTOR (p-mTOR) (Ser2448) (1:1000, CST, #5536), E-cadherin (1:1000, CST, #3195), Vimentin (1:1000, Abcam, ab137321), Fibronectin (1:1000, Abcam, ab2413), ZO-1 (1:1000, Thermofisher, 339188), Occludin (1:1000, Thermofisher, 331594), and β-actin (1:5000, CST, #4970) overnight at 4 °C. After exposing to horseradish peroxidase (HRP)-conjugated secondary antibodies, proteins were visualized using SuperSignal™ West Pico PLUS (Thermofisher Scientific). Protein bands were visualized using a Chemidoc chemiluminescence imaging system (Bio-Rad, Hercules, CA, USA) and quantitative densitometry analysis was performed on the bands of protein detected by immunoblot using Image Lab software (Bio-Rad, Hercules, CA, USA) and normalized to β-actin which served as a loading control.

### 3.8. High Content Image Analysis

Cells were fixed in 2% formaldehyde for 40 min and further incubated with 0.25 mg/mL NaBH4 for an additional 40 min. Samples were exposed to primary antibodies against E-cadherin (1:250), Fibronectin (1:250), and ZO-1 (1:200) for two days at 4 °C. After blocking non-specific binding using 5% BSA for 1 h, visualization was done using secondary antibodies conjugated with Alexa Fluor 594 (1:1000, Thermofisher Scientific, Waltham, MA, USA, A27016). F-actin was stained with Alexa Fluor 488 phalloidin (1:1000, Invitrogen, Waltham, MA, USA A12379). Samples were counterstained with DAPI (1:1000, Thermo Scientific, H3570) and subjected to confocal microscopy (LSM 780, Carl Zeiss, Oberkochen, Germany).

### 3.9. Cytokine Assay

The expression levels of cytokines and chemokines were analyzed using a Human Cytokine Antibody Array (C5) (RayBiotech, Peachtree Corners, GA, USA). Following the manufacturer’s instructions, antibody-embedded membranes were incubated with 1 mL of CM at 4 °C overnight, followed by incubation with HRP-streptavidin at room temperature. Proteins were then visualized using a Chemidoc chemiluminescence imaging system (Bio-Rad, Hercules, CA, USA) and chemiluminescent substrate reagent. The signal intensities were quantified using Image Lab software (Bio-Rad, Hercules, CA, USA).

### 3.10. Drug Penetration Assay

DOX was used to evaluate drug accumulation in spheroids due to its fluorescent properties. After one day of culture, media was replaced with DOX-containing media (300 µM). After 12 h of exposure, the 3D cultured cells were washed with PBS before imaging to remove background fluorescence noise. Optical sections were acquired at 10 µm intervals and stacked into a Z-projection from which fluorescence intensity was calculated. To quantify DOX penetration, the total cell areas with red fluorescence in the 1 mm diameter ROI were calculated in both ASM and CSM at the middle optical section (110 µm). The red fluorescence cell area of CSM and ASM were normalized with respect to the area of the 1 mm ROI, as shown in Figure 6.

## 4. Conclusions

We have successfully optimized 3D cell culture methods and created spheroids by aggregating cells in Matrigel attached on the surface of a 96-pillar plate. According to the initial cell position in Matrigel, 3D-HCC cancer in vitro model (aggregated spheroid model, ASM) was established. The 3D-HCC cells cultured in the ASM showed high resistance to six anticancer drugs that were related to the high expression of cancer proliferation, ECM/tight junctions, and epithelial proteins. Additionally, we found that ASM secreted more cytokines related to ECM regulation and cancer interaction and showed lower drug penetration compared to CSM. Finally, the proposed ASM can mimic TME, including ECM-cell interactions and cell-cell interactions involved in drug resistance and cellular signaling processes. Thus, it may provide more informative data during in vitro drug screening assays of tumor cells and improve the prediction of effective drugs in HCC patients.

## Figures and Tables

**Table 1:** Summary of drug information and quantitatively analyzed drug response results (IC_50_).

		HepG2			Hep3B		
Drug	Target	2D-HTS	CSM	ASM	2D-HTS	CSM	ASM
Sorafenib	VEGFR2-3, PDGFR-β, Raf-1, B-Raf	39.06	28.45	39.89	20.1	18.56	28.65
Cabozantinib	VEGFR2, c-Met	5.65	8.11	16.08	4.79	6.99	10.35
Lenvatinib	VEGFR1-3, FGFR1-4, PDGFRα	34.94	21.77	>100	1.443	>100	>100
Regorafenib	VEGFR1-3, PDGFRβ, Kit, RET, Raf-1, B-RAF	40.47	44.79	48.58	24.77	25.88	72.86
5-FU	DNA/RNA synthesis inhibitor	1.49	1.78	>100	7.71	14.5	87.6
DOX	DNA damage, AMPK	0.052	0.115	1.305	0.029	0.061	0.188

## Supplementary Materials

The following are available online, Figure S1: Quantitative analysis of the 3D Cell forming uniformity.
