# Supplementary figures and images for: Three-Dimensional Aggregated Spheroid Model of Hepatocellular Carcinoma Using a 96-Pillar/Well Plate
Source: Molecules. 2021 Aug 16;26(16):4949. doi: 10.3390/molecules26164949 (PMC8399878; doi:10.3390/molecules26164949)

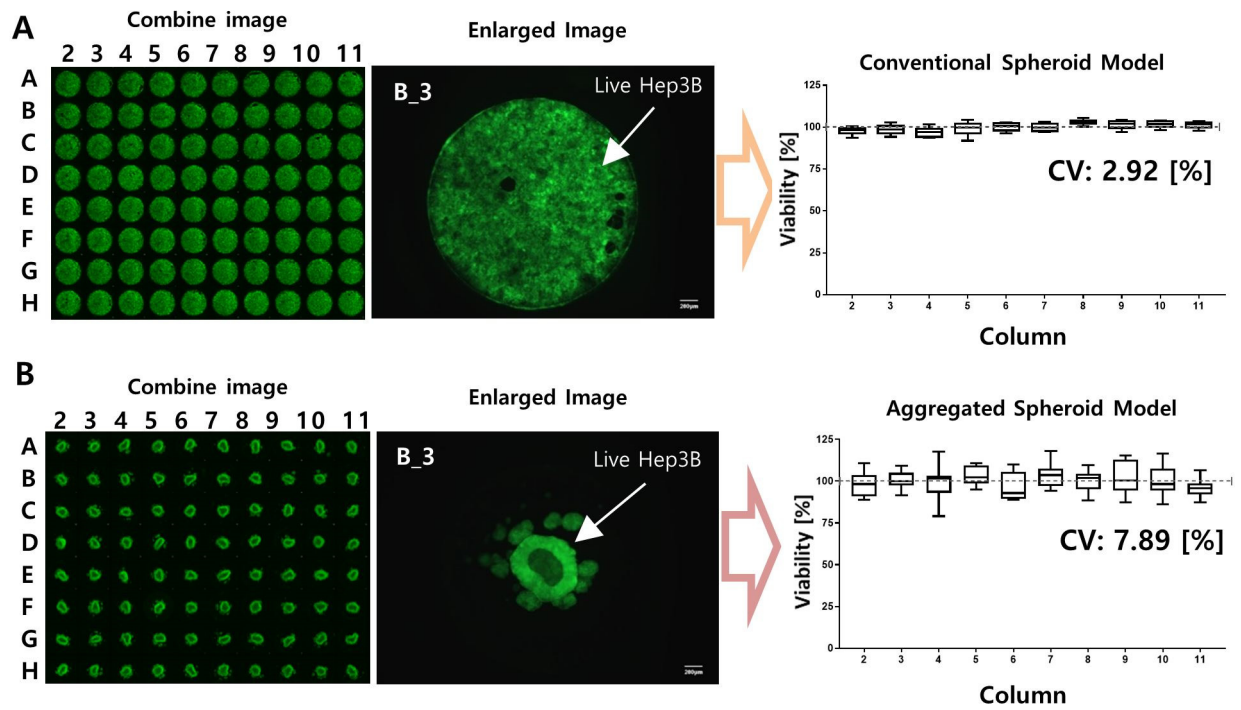

Supplement: Supplementary file 1 [file molecules-26-04949-s001.zip › molecules-1317020-supplementary.pdf]
